# Optimizing second-line endocrine-based treatment in HR positive HER2 negative metastatic breast cancer: a comprehensive expert statement from the Gulf Cooperation Council Region

**DOI:** 10.3389/fonc.2025.1706670

**Published:** 2026-01-22

**Authors:** Ahmed Alshehri, Abdullah Khalaf Altwairgi, Abdulwahab AlTourah, Ahmed Alwbari, Aref Chehal, Francois Calaud, Hashem Al-Hashem, Husam Marashi, Sherif Elsamany, Syed Hammad Tirmazy

**Affiliations:** 1 King Khaled National Guard Hospital, Jeddah, Saudi Arabia; 2 King Fahad Medical City, Riyadh, Saudi Arabia; 3 Kuwait Cancer Control Centre (KCCC), Kuwait City, Kuwait; 4 Almoosa Hospital, Alahsa, Saudi Arabia; 5 Sheikh Shakhbout Medical City (SSMC), Abu Dhabi, United Arab Emirates; 6 National Center for Cancer Care & Research (NCCCR), Doha, Qatar; 7 King Khalid University Hospital, King Saud University, Riyadh, Saudi Arabia; 8 Tawam Hospital, Al Ain, United Arab Emirates; 9 King Abdullah Medical City, Makkah, Saudi Arabia; 10 Dubai Hospital, Dubai, United Arab Emirates

**Keywords:** endocrine therapy resistance, GCC oncology, HR+/HER2-negative mBC, second-line therapy, targeted therapy

## Abstract

Optimizing second-line therapy for hormone receptor-positive (HR+) and human epidermal growth factor receptor 2–negative (HER2-negative) metastatic breast cancer (mBC) in the Gulf Cooperation Council (GCC) is challenged by variations in diagnostic capacity, drug accessibility, comorbidities, and treatment pathways compared with other regions. While international guidelines provide an overarching evidence framework for breast cancer management, their practical application at the regional level often requires adaptation to local healthcare resources. There is an unmet need to optimize the treatment sequencing strategies for patients with HR+/HER2-negative mBC in the GCC region through expert guidance. Given this context, a virtual advisory board involving 10 oncologists from the GCC region was convened in November 2024. The panel aimed to review current evidence and develop pragmatic, implementable recommendations for second-line management. This consensus uniquely contextualizes global evidence for GCC-specific healthcare constraints, addressing gaps in diagnostic access, affordability, and real-world feasibility, while providing treatment recommendations that help clinicians refine therapeutic strategies and incorporate patient preferences for improved outcomes. The panel recommends early genomic testing (PIK3CA, AKT, BRCA, ESR1) to guide therapy, prioritizing targeted agents such as oral SERDs and PI3K/AKT inhibitors in second-line sequencing, cautious use of alpelisib in diabetic patients, and incorporating patient preferences through shared decision-making and multidisciplinary care.

## 1 Introduction

Breast Cancer (BC) represents the most frequent malignancy and the second leading cause of cancer-related fatalities among women in the Gulf Cooperation Council (GCC) region (1). Hormone receptor-positive (HR+) human epidermal growth factor receptor 2 negative (HER2-negative) BC represents the most common molecular subtype, accounting for approximately 70% of all BC cases, globally (2). In the GCC region, this subtype similarly constitutes a high proportion of tumors and is characterized by younger age at presentation and a rising incidence, as reported in regional cancer registries and multicountry GCC analyses (3, 4). In a recent study from the United Arab Emirates (UAE), nearly 66% of the 94 patients diagnosed with BC between 2016 and 2018, were found to have HR+ status (5). Another study involving 78 Arab women with BC during 2010 to 2018 revealed that 69.2% of the cases were positive for estrogen receptors and 65.4% for progesterone receptors, respectively (6). Currently, there is limited data available regarding the prevalence and clinical characteristics of HER2-negative BC in the GCC region. This information is crucial for developing region-specific diagnostic and treatment strategies. This subtype may require tailored therapeutic approaches, particularly as resistance to first-line therapies becomes more prominent.

Advances in targeted therapies have expanded second-line options, yet resistance after cyclin-dependent kinase 4 and 6 (CDK4/6) inhibition remains a significant challenge in decision-making, necessitating the development of effective second-line treatment strategies (7, 8). Newer treatment options including oral selective estrogen receptor degraders (SERDs), targeted agents like inhibitors of phosphatidylinositol-3-kinase (PI3K)/mammalian target of rapamycin (mTOR)/Ak strain transforming (AKT) pathway, and antibody-drug conjugates like sacituzumab govitecan and trastuzumab deruxtecan have become available. Recent data further inform their role in second-line therapy: the EMERALD trial showed benefit with elacestrant (9, 10), and the SERENA-2 trial demonstrated improved activity of camizestrant compared with fulvestrant (11). TROPiCS-02 demonstrated an overall survival benefit with sacituzumab govitecan versus chemotherapy in endocrine-resistant HR+/HER2– metastatic breast cancer (12), and DESTINY-Breast06 reported superior progression-free survival for trastuzumab deruxtecan compared with chemotherapy in HER2-low or HER2-ultra-low HR+ mBC (13).While these targeted therapies have led to improved patient outcomes and delayed the time to start chemotherapy, the optimal sequencing strategy for these agents in second-line treatment remains undefined, highlighting the importance of refining treatment approaches for better long-term efficacy.

In the GCC region, like in many developing countries, there is a reliance on international guidelines for BC management but there are significant disparities in healthcare infrastructure, treatment protocols, genetic heterogeneity, patient characteristics, and access to advanced treatments. These disparities point to the unmet need to optimize the treatment strategies for patients with HR+/HER2-negative mBC through expert guidance. These guidelines can provide critical guidance in prioritizing available diagnostic and therapeutic modalities specific to the regional context.

The GCC-specific adaptations reflect regional epidemiology and healthcare context. The region also has a high burden of metabolic comorbidities, particularly diabetes, which is clinically relevant for therapies such as alpelisib (14). Pharmacogenomic data in the GCC remain limited, with no large-scale sequencing datasets covering all relevant biomarkers (15). Therefore, our recommendations rely on available epidemiologic evidence, expert consensus, and health-system considerations, while highlighting the need for region-specific genomic research.

The present expert opinion article aims to discuss the currently evolving second-line treatment landscape for HR+/HER2- negative mBC in the GCC region, particularly in light of new emerging treatment options. The experts proposed evidence-based treatment recommendations in the second-line setting, tailored to the region’s healthcare resources, ensuring clinical decisions are aligned with the local healthcare resources while optimizing patient outcomes. While international guidelines inform the clinical evidence base, this consensus statement extends their applicability by incorporating region-specific factors, such as diagnostic availability, drug access, insurance constraints, and real-world feasibility in the GCC, to address clinical decision-making challenges not captured in global guidance.

## 2 Methodology

An expert panel of 10 oncologists, from 4 different GCC countries (Kingdom of Saudi Arabia [n=5], Kuwait [n=1], UAE [n=3], Qatar [n=1]) congregated virtually in November 2024. The panel discussed the evolving diagnostic landscape of HR+/HER2-negative mBC by evaluating the clinical utility of emerging biomarkers, identifying the current challenges and barriers to the adoption of testing strategies and their integration into routine clinical practice in GCC region. The aim was also to gain insights into the evolving treatment paradigm on the future role of ET (Endocrine Therapy) in HR+/HER2-negative mBC specifically in the second-line setting. The panel discussed the management practices along with associated regional challenges. They provided strategic as well as implementable recommendations to enhance biomarker testing, treatment strategies and management of treatment-related toxicities associated with the novel treatment options in HR+/HER2-negative mBC in the GCC region.

A structured questionnaire (**Supplementary file**) was disseminated to key experts before the advisory board meeting to gain insight into current challenges encountered in integrating biomarker testing in their routine clinical practice and optimizing the second-line treatment options for HR+/HER2-negative mBC. The compiled responses formed the framework for a comprehensive discussion, guiding the flow of deliberations during the meeting. Recommendations were finalized through iterative discussion and informal consensus, with agreement achieved when the majority of panelists supported the proposed approach. Divergent views were resolved through open dialogue. These recommendations are primarily expert-opinion–based, informed by evidence from pivotal randomized trials and supported by real-world data where available; however, the panel did not formally assign ESCAT (ESMO Scale for Clinical Actionability of molecular Targets) or NCCN (National Comprehensive Cancer Network) evidence levels to each recommendation. We present an expert opinion manuscript with recommendations for optimizing the second-line treatment strategies for HR+/HER2-negative mBC.

## 3 Mechanism of endocrine resistance

While ET remains the mainstay treatment of HR+/HER2-negative mBC, malignant tumors often acquire new mutations or genetic alterations, leading to resistance to ET over time. Various *de-novo* or acquired resistance mechanisms have been implicated, including dysregulated cell cycle regulation, activation of the PI3K/AKT/mTOR pathway, and alterations in the ER (estrogen receptor) pathway (ESR1 tumor mutations) (16).

### 3.1 Loss of estrogen receptor expression

Primary (*de-novo*) resistance to ET is mainly caused by the loss of ER expression (conversion of ER+ to ER−), which occurs in approximately 10% to 20% of the cases (17). These tumors are not responsive to estrogen-driven therapies such as aromatase inhibitors (AIs). Performing biopsy of metastatic tissue at disease progression to evaluate ER status and to guide treatment decisions is crucial.

### 3.2 Mutations in the estrogen receptor (ESR1)

Activating ESR1tumor mutations, reported in approximately 40% of the patients treated with AIs, are the key drivers of secondary ET resistance in patients with HR+/HER2-negative BCs (18). In the SoFEA and EFECT trials, progression-free survival (PFS) with fulvestrant was comparable between patients harboring ESR1 mutations and those with wild-type tumors, whereas a reduction in PFS associated with ESR1-mutant disease was more apparent with exemestane (19). In PALOMA-3, fulvestrant plus palbociclib demonstrated consistent progression-free and overall survival benefits, irrespective of ESR1 mutation status (20, 21).

### 3.3 Activation of alternative signaling pathways

Activating mutations in the *PIK3CA* represent the most frequent mutation type in the advanced HR+/HER2-negative mBC, accounting for nearly 40% of the tumors (22, 23). Recent evidence suggests that these mutations are most commonly reported in the hotspots of H1047R, E545K, E542K, N345K, and H1047L (24–26). Dysregulated signaling through the PI3K pathway is associated with tumorigenesis, disease progression, and resistance to therapy (27). Patients presenting tumors harboring PIK3CA mutations have poor outcomes and are less sensitive to chemotherapy, compared to those with wild-type PIK3CA (23). Additional tumor alterations may include mutations in AKT1 and phosphatase and tensing homolog (PTEN) signaling pathways. Activating AKT1 mutations are present in approximately 7% of the HR+ HER2-negative BC cases, E17K being the most commonly reported alteration, leading to constitutive signaling activation through aberrant localization to the plasma membrane (28). PTEN is the negative regulator of the PI3K pathway, and its loss results in the activation of downstream AKT/mTOR signaling (29). Further, mutations in components of the mitogen-activated protein kinase (MAPK) pathway, such as NF1, KRAS, HRAS, BRAF, ERBB2, and EGFR, have been linked to shorter duration of response and occur independent of ESR1 mutations (28). As a result, therapeutic strategies targeting RAS and other key oncoproteins in the MAPK pathway (RAF, ERK, and MEK) hold the potential to overcome endocrine resistance caused by these pathway alterations.

Additional pathological mutations include acquired alterations related to HER2, BRCA, TP53, and other genes responsible for driving tumor progression and therapy resistance. Tumor environment in endocrine resistance encompassing various cellular components, including immune cells, stromal cells, blood vessels, and extracellular matrix, also contribute to endocrine resistance (17).

## 4 Role of genomic profiling in treatment decisions

Genomic and molecular profiling helps identify actionable mutations and alterations that can guide the choice of subsequent treatments, improving clinical outcomes (27). Identification of PIK3CA mutations allows the use of targeted therapies like alpelisib (a PI3K inhibitor) in combination with letrozole (30). The phase III SOLAR-1 trial demonstrated that the addition of alpelisib to fulvestrant significantly improved PFS in patients with PIK3CA-mutated HR+/HER2-negative mBC, compared to fulvestrant alone (31, 32).Additionally, this finding highlights the importance of targeting the PI3K pathway in the second-line setting.

Moreover, genomic profiling aids in identifying alterations that may influence resistance mechanisms, such as AKT or PTEN mutations, both of which are relevant in selecting combination therapies (33). These alterations can impact the effectiveness of treatments like CDK4/6 inhibitors and guide the use of targeted therapies. In the CAPItello-291 trial, the addition of capivasertib (AKT inhibitor) to fulvestrant resulted in clinically meaningful improvement in PFS in patients with tumors harboring PIK3CA/AKT1/PTEN mutations (34).Therefore, tumors in patients with HR+ aBC (advanced breast cancer) harboring PIK3CA/AKT mutations and/or PTEN loss may benefit from combining capivasertib with fulvestrant.

Detection of ESR1 tumor mutations is crucial as it may guide the early transition from an AI-based regimen to fulvestrant, thus optimizing clinical outcomes (35). Evidence suggests that patients with ESR1 tumor mutations receiving fulvestrant plus CDK4/6 inhibitors in the first-line demonstrate poor outcomes (36). Additionally, phase III EMERALD trial, which demonstrated improved PFS with the oral SERD elacestrant in patients with ESR1 tumor mutations. Based on these findings, the American Society of Clinical Oncology (ASCO) has updated its guidelines to recommend routine ESR1 mutation testing to guide treatment decisions in patients with HR+/HER2-negative mBC (37). This revision emphasizes the importance of testing for ESR1 tumor mutations to tailor treatment with therapies like elacestrant, which have shown efficacy in overcoming resistance to standard hormonal therapies. On the other hand, the European Society for Medical Oncology (ESMO) guidelines consider ESR1 tumor mutation testing as optional (ESCAT II-A), acknowledging its potential role in treatment selection (7). This distinction reflects the evolving understanding of genomic profiling and its impact on therapy decisions, with a growing consensus around personalized treatment strategies.

In a recent study, nearly half of the patients were identified with level 1 actionable genetic mutations (ESCAT level of evidence I/II) using prospective genomic profiling. Identification of BRCA1/2 mutations suggests the need for germline testing, which can determine eligibility for poly (ADP-ribose) polymerase (PARP) inhibitors. Notably, the study used targeted sequencing panel covering fewer genes than some commercially available assays, indicating that a smaller panel (focused only on ESCAT LOE I/II genes) would be sufficient for most patients who could benefit from genomic testing in daily practice (38).

Tissue biopsies are traditionally used to characterize metastatic breast cancer (mBC) but are limited by spatial heterogeneity and invasiveness. They help identify mutations like PIK3CA, ESR1, and AKT, which are important for guiding second-line therapy (39). Liquid biopsies are less invasive alternative, analyze circulating tumor DNA (ctDNA) from blood samples, and can track mutations like ESR1 to guide treatment decisions, such as switching from AIs to fulvestrant. However, liquid biopsy may be less sensitive than tissue biopsy and can produce false negatives, particularly when ctDNA levels are low. A retrospective analytical comparison of tissue and liquid biopsy next-generation sequencing (NGS) data revealed a high positive percent agreement (PPA) for PIK3CA/AKT1/PTEN alterations in cases with ctDNA tumor fraction (TF) ≥1% (92.5% for TF ≥10% and 97.1% for TF 1% to 10%). However, detection rate for samples with TF <1% was found to be lower (PPA 33.9%) (40). A large-scale United States based study analyzing data from approximately 280 cancer clinics (2011 to 2023) found that the tumors of around 60% of patients exhibited at least one genetic alteration in the first-line of therapy. Notably, ESR1 mutations were present in 8.1% of tissue biopsies and 17.5% of liquid biopsies with a TF of ≥1%. Conversely, rates of PTEN loss were higher with tissue biopsy (4.3%) than liquid biopsy (1% in TF ≥ 1%) (36). Another study examining the concordance between tumor tissue and ctDNA for the detection of oncogenic mutations found high concordance for PIK3CA and AKT1, moderate concordance for ESR1, and low concordance for PTEN. Moreover, a higher number of ESR1 alterations were detected in ctDNA, while PTEN alterations were more commonly found in tumor tissue, suggesting the emergence of acquired ESR1 tumor mutations after estrogen deprivation and highlighting the current limitations of ctDNA assays in detecting PTEN copy number loss (41). These findings suggest the clinical relevance of comprehensive genomic profiling with liquid biopsy for identification of acquired mutations in the second-line.

NGS is a powerful tool that allows for the simultaneous analysis of multiple genes, providing a comprehensive profile of a tumor’s genetic makeup. NGS can detect a wide range of mutations, including PIK3CA, ESR1, AKT, and PTEN and particularly ESR1 mutations, which are associated with resistance to AIs, and in selecting targeted therapies such as elacestrant or alpelisib or capivasertib.

## 5 Optimizing second-line endocrine-based treatments

### 5.1 Sequencing strategies

For patients who progress on first-line CDK4/6 inhibitor plus ET, the presence of molecular alterations influences second-line selection. In patients with ER+ mBC, ESR1 tumor mutations have been associated with resistance to AI therapy, accounting for nearly 40% of the acquired resistance to AIs in metastatic settings (42, 43). The frequency of ESR1 tumor alterations tends to increase as the disease progresses through successive lines of treatment, leading to poorer clinical outcomes in patients with HR+ mBC (19, 39, 44). Notably, in the MAINTAIN trial, patients with detectable ESR1 tumor mutations had less benefit from ribociclib plus fulvestrant versus fulvestrant alone (45). Similarly, patients with ER+ BC and germline BRCA mutations may experience a shorter PFS with first-line CDK4/6 inhibitors (46) potentially due to allelic imbalance in RB1 or loss of heterozygosity, which may serve as a potential mechanism of resistance to CDK4/6 inhibition in germline BRCA2 carriers (47). Genomic alterations within the PI3K/AKT/mTOR pathway are found in about 50% of patients with HR+/HER2-negative mBC (48, 49), and are recognized as key drivers of resistance to ET and disease progression. Studies have highlighted the correlation between the PI3K/AKT/mTOR pathway and resistance to CDK4/6 inhibitors (50–52). Understanding these resistance mechanisms is crucial in guiding second-line treatment choices and determining whether a patient remains sensitive to further endocrine-based therapy or requires a shift to targeted therapies or chemotherapy based approaches (53, 54).

### 5.2 Monotherapy versus combination approaches

The choice between combination therapy or monotherapy depends on multiple factors, including prior treatment exposure, disease burden, patient preferences, and tolerability considerations.

The fulvestrant monotherapy demonstrated limited benefit in patients who have progressed after receiving CDK4/6 inhibitors plus AI, across several randomized trials (9, 45, 55) and is not typically recommended as a single-agent treatment after progression on CDK4/6 inhibitors combined with AI (56, 57). In patients with HR+/HER2-negative mBC without targetable tumor mutations and who had a prolonged exposure or slower progression on first-line CDK4/6 inhibitors with ET, a re-challenge with a different CDK4/6 inhibitor may be a viable option (58). The MAINTAIN trial showed significant improvement in PFS when patients were treated with ribociclib after prior CDK4/6 inhibitor plus ET therapy. In contrast, the PACE and PALMIRA trials showed no benefit in terms of PFS or overall survival (OS) when continuing palbociclib beyond progression, indicating that switching CDK4/6 inhibitors may be more effective due to distinct resistance mechanisms (59, 60). Further, results from the postMONARCH trial demonstrated the clinical benefit of continuing CDK4/6 inhibition with abemaciclib, combined with fulvestrant, following disease progression on a previous CDK4/6 inhibitor (**Table 1**) (49).

**Table 1:** Summary of evidence from pivotal clinical trials in patients with HR+ HER2-negative Mbc.

Treatment dtrategy	Trial name	Study population	Treatment arms (n)	Median PFS (months, 95% CI)	Hazard ratio (95% CI); P-value
ET + CDK4/6 continuation	MAINTAIN (45)	Progression on ET + CDK4/6; switch ET	Palbociclib (n = 103)	2.8 (2.7–3.3)	0.6 (0.4–0.9); *P* = 0.006
			Switch to ribociclib (n = 14)	5.3 (3.0–8.1)	
	PACE (59)	Progression on CDK4/6 + AI	Fulvestrant (n=72)	4.8	1.11 (90% CI, 0.79–1.55); P = 0.62
			Fulvestrant + Palbociclib (n=73)	4.6	
			Fulvestrant + Palbociclib + Avelumab (n=75)	8.1	0.75 (90% CI, 0.50–1.12); P = 0.23 vs Fulvestrant
	PALMIRA (60)	Progression on first-line Palbociclib + ET	Palbociclib + ET (n=136)	4.9 (3.6–6.1)	0.84 (0.66–1.07); P = 0.149
			ET alone (n=62)	3.6 (2.5–4.2)	
	PostMONARCH (49)	Progression on first-line CDK4/6 + Fulvestrant	Abemaciclib + Fulvestrant (n=182)	6.0 (5.6–8.6)	0.73 (0.57–0.95); P = 0.017
			Placebo + Fulvestrant (n=186)	5.3 (3.7–5.6)	
ET + mTOR inhibitor	BOLERO-2 (61)	Progression on non-steroidal AI	Exemestane + Everolimus (n=485)	7.8	0.45 (0.38–0.54); P<0.0001
			Placebo + Exemestane (n=239)	3.2	
ET + PI3K inhibitor	SOLAR-1 (62)	PIK3CA-mutant, post-AI	Alpelisib + Fulvestrant (n=169)	11.0 (7.5–14.5)	0.65 (0.50–0.85); P<0.001
			Placebo + Fulvestrant (n=172)	5.7 (3.7–7.4)	
	BYLieve (32)	PIK3CA-mutant, post-CDK4/6	Alpelisib + Fulvestrant (n=127)	8.0 (5.6–8.6)	
			Alpelisib + Letrozole (n=126)	5.6 (3.7–7.1)	
ET + AKT inhibitor	CAPItello-291 (39)	Post-AI ± CDK4/6; PIK3CA/AKT1/PTEN-altered subgroup	Capivasertib+fulvestrant (n = 155)	7.3 (5.5–9.0)	0.50 (0.38–0.65); *P* < 0.001
			Placebo + Fulvestrant (n=134)	3.1 (2.0–3.7)	
	FAKTION (63)	Progression on AI	Capivasertib + Fulvestrant (n=69)	10.3 (5.0–13.4)	0.56 (0.38–0.81); P = 0.0023
			Placebo + Fulvestrant (n=71)	4.8 (3.1–7.9)	
Oral SERD	EMERALD (9)	ESR1-mutant, post-CDK4/6; ≤ 1 CT	Elacestrant (n=115)	3.8	0.55 (0.39–0.77); P = 0.0005
			Standard ET (n=113)	1.9	
PARP inhibitors	OlympiAD (64)	Germline *BRCA*-mutant HER2-negative mBC; ≤2 CT lines	Olaparib (n=205)	7.0	0.58 (0.43–0.80); P<0.001
			Physician’s choice CT (n=97)	4.2	
	EMBRACA (65)	Germline *BRCA*-mutant HER2-negative mBC	Talazoparib (n=287)	8.6	0.54 (0.41–0.71); P<0.001
			Standard CT (n=144)	5.6	

AI, aromatase inhibitor; BRCA, breast cancer gene; CDK, cyclin-dependent kinase; CI, confidence interval; CT, chemotherapy; ER, estrogen receptor; ESR1, estrogen receptor 1; ET, endocrine therapy; HER2, human epidermal growth factor receptor 2; HR, hormone receptor; mBC, metastatic breast cancer; mTOR, mammalian target of rapamycin; PARP, poly (ADP-ribose) polymerase; PFS, progression-free survival; PIK3CA, phosphatidylinositol-4, 5-bisphosphate 3-kinase catalytic subunit alpha; AKT, protein kinase B (a key downstream effector in the PI3K pathway).

Combination therapies including ET with targeted therapies, such as alpelisib and capivasertib, have shown potential in overcoming resistance in HR+/HER2-negative mBC, as evident from the findings of PALOMA-3 and MONARCH 2 trials (7, 58). Similarly, for patients with PIK3CA, PTEN or AKT alterations, AKT inhibitors like capivasertib in combination with fulvestrant is recommended (58). The SOLAR-1 trial showed that combining alpelisib with fulvestrant significantly prolonged PFS in patients with PIK3CA-mutated, HR+/HER2-negative mBC who had progressed on prior ET (39). Similarly, the BYLieve trial demonstrated clinical benefit with this combination, especially in patients who progressed on CDK4/6 inhibitors plus AI (66). Additionally, the CAPItello-291 (39) and FAKTION trials (63) demonstrated the efficacy of capivasertib plus fulvestrant for tumors in patients with HR+ mBC harboring PIK3CA/AKT1/PTEN alterations who had progressed on prior AI therapy, with or without CDK4/6 inhibitors (**Table 1**). The EMERALD trial showed that elacestrant significantly improved PFS compared to standard ET in both the overall and ESR1 tumor mutated populations. For patients with PIK3CA/AKT1/PTEN alterations and ESR1 tumor mutations, capivasertib plus fulvestrant or elacestrant monotherapy are preferred treatment options, respectively (**Table 1****) (**9). More recently, the EMBER-3 trial showed significant improvement in PFS with imlunestrant compared to standard ET in the patients with tumors harboring ESR1 mutations (5.5 versus 3.8 months, respectively) but not in the overall population (Hazard Ratio, 0.87; 95% CI, 0.72−1.04; *P* = 0.12). Imlunestrant in combination with abemaciclib resulted in further enhancement in PFS compared to imlunestrant monotherapy, irrespective of the status of ESR1 mutations (9.4 versus 5.5 months, respectively; HR, 0.57; 95% CI, 0.44−0.73; *P* < 0.001) (67).

The OlympiAD and EMBRACA trials demonstrated that PARP inhibitors (olaparib and talazoparib) significantly improve PFS in HER2-negative mBC patients compared to standard chemotherapy. These findings support their use as a viable treatment for patients with germline BRCA1/2 mutations. Additionally, PARP inhibitors may also benefit those with germline PALB2 mutations (**Table 1**) (64, 65, 68, 69).

The BOLERO-2 study showed that everolimus plus exemestane significantly improved PFS in HR+/HER2-negative mBC patients who progressed on AIs, though no OS benefit was observed. The BOLERO-6 trial confirmed that everolimus plus exemestane provided a PFS benefit over everolimus monotherapy, supporting its use in endocrine-based therapy sequences. For patients unable to tolerate this combination, capecitabine offers a viable alternative with similar PFS and OS outcomes (**Table 1**) (61, 70).

### 5.3 Testing strategies

Testing for mutations such PIK3CA, AKT1, PTEN is recommended at the time of recurrence or progression in patients with HR+/HER2-negative mBC receiving ET (with or without CDK4/6 inhibitors), to determine their eligibility for treatment with capivasertib (PIK3CA/AKT1/PTEN alterations) or alpelisib (only for patients with PIK3CA tumor mutations). Given the positive benefit-risk profile demonstrated by PARP inhibitors, olaparib or talazoparib, in OlympiAD (64) and EMBRACA (65) trials respectively, it is important to consider testing for germline BRCA1/BRCA2 mutations in patients with HER2-negative mBC to identify patients who may be candidates for treatment with the PARP inhibitors.

Although testing for PIK3CA, AKT, and BRCA is recommended at diagnosis, comparative data on predictive performance [e.g., sensitivity, specificity, AUROC (Area Under the Receiver Operating Characteristic Curve), Youden’s J] and assay differences between tissue and ctDNA remain limited. Our recommendations therefore rely on biological rationale, guideline alignment, and therapy eligibility. Potential false positives or negatives, particularly with ctDNA at low tumor fractions, should be considered, and confirmatory testing may be needed when results conflict with clinical context. Prospective validation remains an important future priority (71).

The ASCO and ESMO guidelines both emphasize the importance of routine testing for certain biomarkers and mutations in second-line treatment for HR+/HER2-negative mBC (7, 37). The guidelines recommend testing for the emergence of ESR1 tumor mutations after relapse or progression on ET (administered with or without CDK4/6 inhibitor) in patients with HR+/HER2-negative mBC to guide the decision to use elacestrant (9).

Exploratory biomarker analyses from the phase III ASCENT trial suggest that higher TROP-2 expression may be associated with numerically greater benefit from sacituzumab govitecan; however, clinically meaningful efficacy was observed across low, intermediate, and high expression subgroups. Accordingly, TROP-2 expression has not been validated as a predictive biomarker, and routine testing is not required for treatment selection (72).

### 5.4 Management of adverse events

Despite the advancements in the targeted therapies, the key challenge is the safety profile of the novel drugs, which may impact patient adherence and quality of life (QoL). Both alpelisib (PI3K inhibitor) and capivasertib (AKT inhibitor) cause grade 3 or greater adverse events (AEs) including diarrhea (alpelisib [6.7%] versus capivasertib [9.3%]), rash (alpelisib [9.9%] versus capivasertib [12.1%]), and hyperglycemia (alpelisib [36.6%] versus capivasertib [2.3%]) (39, 73). Alpelisib-induced hyperglycemia may be severe and requires proactive monitoring of glucose levels, with metformin recommended for prevention and has shown to significantly decrease the occurrence of grade 3 or higher hyperglycemia in the phase II METALLICA (74) study.

Additionally, newer targeted therapies like elacestrant (a SERD) cause most common gastrointestinal AEs like nausea (35%) and vomiting and fatigue (19% each) (9, 10). Olaparib reported AEs of anemia (40.0%), neutropenia (27.3%), nausea (58.0%), vomiting (32.2%), fatigue (29.8%), diarrhea (20.5%) and hematologic AEs (75). Management includes antiemetics for nausea, blood transfusions, growth factors or dose modifications for anemia and neutropenia and anti-diarrheal medications for diarrhea. Fatigue can be managed through dietary changes, improved sleep, psychosocial support and psychostimulants as required (76, 77).

Several clinical trials have evaluated patient reported outcomes with the novel targeted agents. For instance, the Global Health Status (GHS)/QoL and functional status were found to be maintained from baseline among the patients treated with alpelisib+ fulvestrant in the SOLAR-1 trial. Moreover, the time to deterioration (TTD) of 10% in GHS/QoL and functional status was comparable to placebo group (62). In the CAPItello-291 trial, the addition of capivasertib to fulvestrant not only improved PFS but also demonstrated delayed deterioration in patient reported QoL (39), a finding that contrasts with the QoL outcomes observed with alpelisib. While there was no clinically meaningful difference in functional and symptoms scales for elacestrant and SoC groups in the EMERALD trial, the incidence of very severe nausea (4% versus 14.3%, respectively) and severe vomiting (9.1% versus half, respectively) was relatively lower with elacestrant, compared to SoC, along with numerically better outcomes for self-care, mobility, and usual activities (78). Further, the OlympiAD trial showed statistically significant improvements in GHS/QoL from baseline with olaparib (*P* = 0.0035) and TTD (HR, 0.44 [0.25-0.77; *P* = 0.004], however, nausea/vomiting symptom score was worse in the olaparib group (79). Aligned with these results, talazoparib outperformed chemotherapy, with significant benefit in terms of GHS/QoL scores, TTD, and multiple cancer-related symptoms and functions (80).

Results from a German study exploring patient preferences for the treatment of locally advanced or mBC, revealed that QoL (defined as physical mobility and agility) was identified as the key determining factor influencing therapeutic decisions in the second-line (utility score of 19.4%), followed by OS (15.2%) and PFS (14.4%) (81). Additionally, a recent multicentric online survey highlighted a disconnect between healthcare providers and patients regarding the importance of QoL discussions and the impact of AEs on treatment outcomes (82). Regular assessment of patient preferences using validated questionnaires or structured interviews may help physicians monitor treatment efficacy and safety profile, thus allowing for timely adjustments if needed (83).

The panel reviewed validated QoL instruments, including EORTC (European Organisation for Research and Treatment of Cancer) QLQ-C30 with the updated QLQ-BR42 breast module, FACT-B (Functional Assessment of Cancer Therapy-Breast), and EQ-5D-5L (EuroQol 5-Dimension, 5-Level). No single tool was formally endorsed for routine GCC practice; instead, centers may select instruments based on feasibility, workflow, and whether utility values are needed for cost-effectiveness analyses (84–87).

## 6 Expert panel recommendations

**Figure 1:**
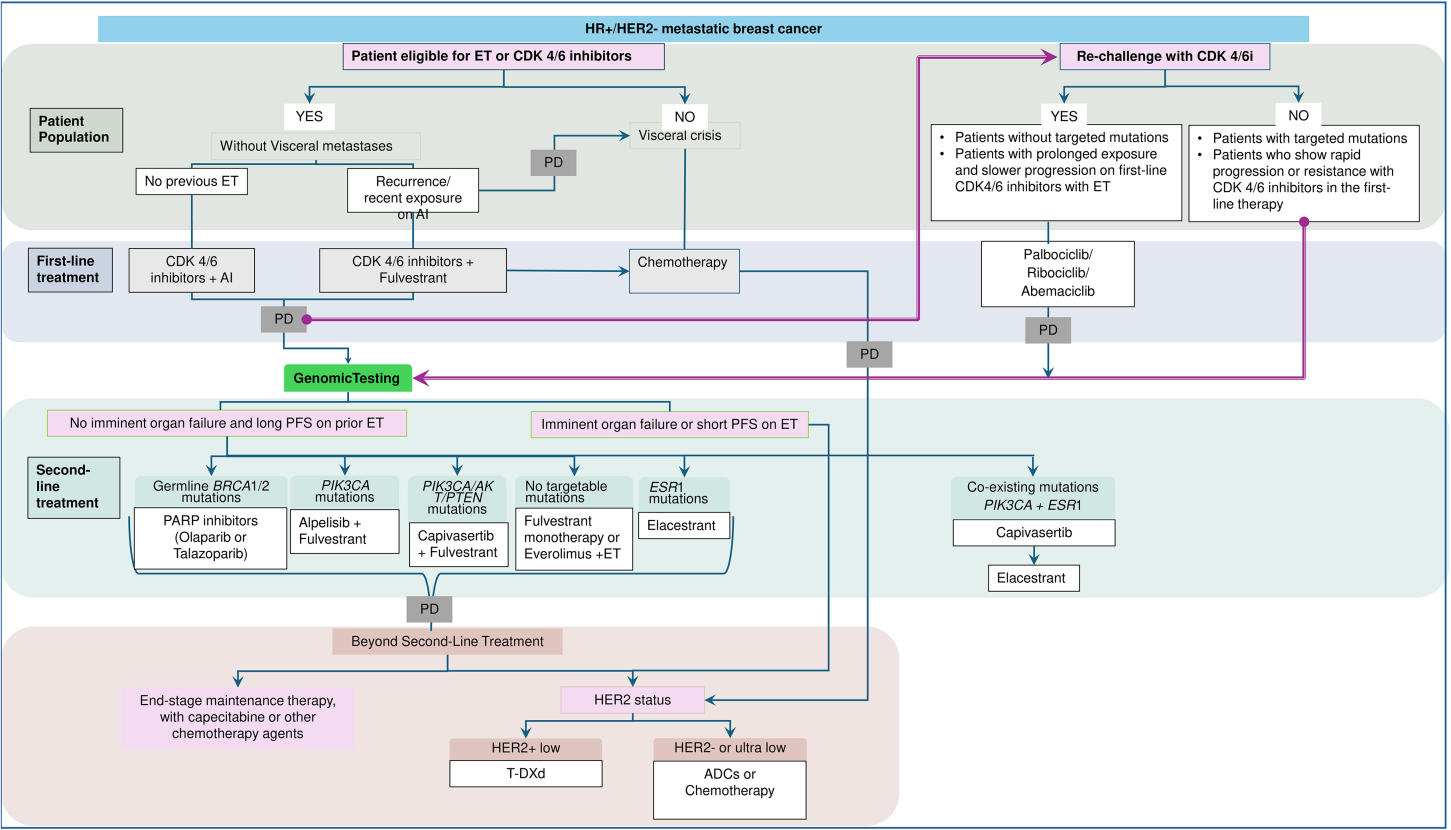
Algorithm for optimal selection of second-line treatment strategies in HR+ HER2-negative mBC. ADC, antibody-drug conjugates; AI, aromatase inhibitor; AKT, Ak strain transforming; BRCA, BReast CAncer gene; CDK, cyclin-dependent kinase; ESR, estrogen receptor; ET, endocrine therapy; HER2, human epidermal growth factor receptor 2; HR, hormone receptor; PARP, poly (ADP-ribose) polymerase; PD, disease progression; PFS, progression-free survival; PIK3CA, phosphatidylinositol-4, 5-bisphosphate 3-kinase catalytic subunit alpha; PTEN, Phosphatase and tensin homolog deleted on chromosome 10; T-DXd, trastuzumab deruxtecan.

Based on the existing literature and clinical practice, the expert panel has delineated practical recommendations for genomic testing (**Box 1**), treatment sequencing in the second-line (**Box 2**; **Figure 1**), and patient-centered approaches (**Box 3**) for effective management of HR+ HER2-negative mBC in the GCC region.

Box 1 **Expert recommendations for genomic testing in the second-line treatment of patients with HR+/HER2-negative mBC**

- Testing for basic biomarkers (e.g., PIK3CA, AKT, BRCA) is recommended at diagnosis which will enable immediate readiness for subsequent treatments if progression occurs, without the need for additional testing delays.
- For testing platforms, liquid biopsy is preferred for detecting mutations such as ESR1 and PIK3CA after progression on first-line therapy, while tissue biopsy is recommended when liquid biopsy results are negative or when the disease burden is low, and no clinical signs of progression are observed.
- Testing for PIK3CA, AKT1, and PTEN mutations at the time of recurrence or upon disease progression on ET (either with or without CDK4/6 inhibitors) to determine their eligibility for treatment with capivasertib (PIK3CA/AKT1/PTEN alterations) or alpelisib (only for patients with PIK3CA tumor mutations).
- Testing for germline BRCA1/BRCA2 mutations at first-line or during adjuvant setting can be considered to identify suitable candidates for treatment with the PARP inhibitors to avoid any delays in treatment decisions.
- Early testing for ESR1 and PIK3CA mutations that may be acquired during treatment is recommended after progression on first-line treatment.
- Testing for the emergence of ESR1 tumor mutations is recommended to guide the decision to use elacestrant after relapses or progression on ET (administered with or without CDK4/6 inhibitor for >12 months).
- Repeat testing of ESR1 tumor mutations could be considered at later lines if a patient is still considered for endocrine treatment.

Box 2 **Summary of expert recommendations for second-line treatment sequencing in patients with HR+/HER2-negative mBC based on common clinical scenarios**

- Combination therapy of CDK4/6 inhibitors and hormonal therapy is the standard first-line approach for patients with HR+/HER2-negative mBC, particularly after progression on initial ET, in patients without imminent organ failure.
- When the disease progresses on AIs and there is visceral metastasis, switching to fulvestrant in combination with CDK4/6 inhibitors is recommended to improve outcomes. Patients who progress early on CDK 4/6 inhibitors (within the first year) may have PIK3CA-dependent tumors and are best targeted with combination of a PI3K inhibitor/AKT inhibitor (e.g., alpelisib/capivasertib) with ET.
- For patients with tumors harboring PTEN or AKT mutations, AKT inhibitors like capivasertib in combination with fulvestrant is recommended.
- In patients with ESR1 tumor mutations with prolonged duration of ET (> 12 months), switching to SERDs such as elacestrant is recommended.
- Patients without targetable tumor mutations who have had prolonged exposure and slower progression on first-line CDK4/6 inhibitors with ET, re-challenge with a different CDK4/6 inhibitor may be considered.
- Patients who show rapid progression or resistance with CDK 4/6 inhibitors in the first-line therapy, re-challenge with other CDK 4/6 inhibitors may not be effective.
- For patients with PIK3CA-mutated tumors who experience progression on a prior ET including an AI, with or without a CDK4/6 inhibitor, treatment with alpelisib plus fulvestrant is preferred. However, careful screening and management of common toxicities with alpelisib like hyperglycemia are required.
- In the Gulf region, where diabetes is prevalent, alpelisib should be used cautiously, with careful blood glucose management and possible referral to a diabetologist.
- Capivasertib has a superior safety profile than alpelisib and can be a preferred treatment option for patients with PIK3CA tumor mutations.
- Capivasertib plus ET is recommended for patients progressing on previous AI therapy (with or without a CDK4/6 inhibitor) with tumors harboring PIK3CA or AKT1 or PTEN alterations.
- In patients with coexisting tumor mutations (ESR1 and PIK3CA), it is preferable to consider capivasertib (AKT inhibitor) to target the PIK3CA mutation, followed by elacestrant. Individualized consideration is required for each case.
- For BRCA-mutated patients, PARP inhibitor monotherapy (olaparib or talazoparib) is considered a viable treatment option. Additionally, it may be considered for individuals with germline PALB2 mutations.
- Patients with visceral metastases (without signs of visceral crisis) can be considered for treatment with the combination of CDK4/6 inhibitor + ET, before resorting to chemotherapy.
- For patients with tumors without relevant mutations, fulvestrant plus everolimus or chemotherapy is to be considered. Fulvestrant monotherapy is usually not recommended due to limited efficacy.
- In patients with continued disease progression following second-line therapy with the combination of targeted agents and CDK 4/6 inhibitor, treatment is maintained at the end-stage, potentially with capecitabine or other chemotherapy agents, depending on the response to treatment.
- For tumors with HER2 low or ultra-low expression, alternative therapies such as antibody-drug conjugates (T-DXd and sacituzumab govitecan) or chemotherapy may be considered. Additionally, these treatment options may be beneficial for patients who show rapid disease progression on CDK 4/6 inhibitor therapy, particularly those experiencing progression within 6 months of initiating first-line ET+CDK 4/6 inhibitor or recurrence ≤24 months following adjuvant ET.

Box 3 **Expert recommendations for patient-centered approaches**

- Engaging patients in shared decision-making by discussing options, risks, benefits, and personal preferences, ensuring their values are incorporated into the treatment plan.
- Developing individualized therapies based on the patient’s genetic profile (e.g., testing for PIK3CA, BRCA, or ESR1 mutations) to ensure the most effective treatment.
- Regular assessments of QoL to ensure that treatment plans align with the patient’s physical, emotional, and social well-being.
- Engaging a multidisciplinary team (oncologists, nurses, psychologists, nutritionists) to address all aspects of the patient’s health and well-being, ensuring a holistic approach to care.

## 7 Access and implementation challenges in the GCC region

### 7.1 Barriers to the availability of targeted therapies and diagnostics

Access to molecular diagnostics in the Gulf region for HR+/HER2-negative mBC faces few challenges and disparities in availability, affordability, and consistency. Many hospitals in the GCC region lack centralized or in-house laboratories for advanced molecular testing like NGS. As a result, these facilities rely on external laboratories funded by pharmaceutical companies or send out samples to international labs for NGS testing. In countries like Qatar, NGS and single-gene testing capabilities are available in major cancer centers. In addition, liquid biopsy tests like Guardant 360 testing (advanced BC) and Guardant Reveal testing (early BC) are provided free of cost to all Qatari nationals, making these tests more accessible. In Saudi Arabia, testing for BRCA, ESR1, and PIK3CA mutations is widely available in public hospitals either through hospital-funded services or through a company that supports testing. However, testing for other mutations like PTEN and AKT is not as readily accessible. In UAE, germline BRCA testing is available in-house at the public sector hospitals with government coverage available for UAE nationals (local patients). In the private sector, molecular testing is covered under insurance coverage; however, some group insurance policies deny coverage, limiting access for certain patients. Another major hurdle is the absence of centralized or in-house laboratories for NGS testing in many hospitals across the GCC region. In Kuwait, patient samples are often handled in-house with available tissue and liquid biopsy NGS platforms, which may decrease the turnaround time. However, access to newer drugs like capivasertib and elacestrant, however, restricts the number of patients who can benefit from targeted therapies.

Published cost-effectiveness analyses (88, 89) show substantial heterogeneity across health systems due to differences in economic assumptions, methodological approaches, and willingness-to-pay thresholds. Based on this, the panel recommends locally adapted budget-impact modeling that incorporates GCC-specific unit costs, EQ-5D–based utility values, and scenario analyses reflecting variations in insurance coverage. Although no formal pharmacoeconomic modeling was performed, the panel recognized cost and coverage constraints as major determinants of real-world feasibility and emphasized aligning treatment selection with local affordability and access considerations.

### 7.2 Strategies to improve access to precision medicine

Reflecting on these challenges, the experts recommended improving accessibility to molecular testing in the region. Establishing a centralized reference laboratory to facilitate the implementation of standardized testing protocols, ensuring greater consistency of results. Additionally, the availability of in-house testing capabilities for PIK3CA, AKT, PTEN and BRCA testing is critical for guiding treatment decisions, streamlining the process, and reducing the turnaround time. Localized testing may help facilitate research initiatives at the national level and contribute to the improvement of clinical effectiveness data. Considering that the PIK3CA mutations tend to remain consistent from first-line to second-line treatments, the ready availability of PIK3CA test results at the time of first-line treatment will help ensure a smooth transition if a change in therapy is required. While there may be instances of acquired mutations during treatment, early testing helps with preparedness and timely adjustments to the treatment plan.

## 8 Limitations

This consensus reflects input from a small expert panel from the GCC and was not developed through a formal guideline methodology or structured evidence-grading system, which may limit its generalizability. Biomarker predictive performance, pharmacoeconomic modeling, and GCC-specific epidemiologic or genomic data could not be systematically assessed due to limited available evidence, so several recommendations rely on expert opinion and real-world feasibility considerations. Prospective regional studies are needed to validate and refine these guidance statements.

## 9 Conclusion

In the evolving therapeutic landscape of HR+/HER2-negative mBC, optimizing second-line treatment requires a strategic approach that enhances survival while maintaining safety and QoL. Multiple factors including disease burden, prior therapy response, treatment duration, and patient characteristics should guide treatment selection and sequencing strategies. Additionally, regional considerations such as drug availability, accessibility, cost, and regulatory approval also influence therapy selection. While current testing for mutations such as BRCA and PIK3CA is essential for personalized treatment, several challenges hinder the effectiveness and accessibility of molecular testing including limited availability of testing platforms for mutations like PTEN, AKT, and ESR1, high costs, and limited insurance coverage. The proposed treatment algorithm may assist clinicians in refining therapeutic strategies while incorporating patient preferences and real-world feasibility for improved outcomes in the second-line treatment strategies for patients with HR+/HER2-negative mBC in the GCC region.
