# Supplementary material for: Optimizing second-line endocrine-based treatment in HR positive HER2 negative metastatic breast cancer: a comprehensive expert statement from the Gulf Cooperation Council Region
Source: Front Oncol. 2026 Jan 22;15:1706670. doi: 10.3389/fonc.2025.1706670 (PMC12872529; doi:10.3389/fonc.2025.1706670)
Supplement: Supplementary file 1 [file DataSheet1.pdf]

GCC Breast Cancer Expert Group Optimizing ET treatment in 2L HR+ve/HER-ve mBC

Pre-meeting Questionnaire

Identification details

**Name:**	
**Specialty:**	☐ Medical Oncologist<br>☐ Surgical Oncologist<br>☐ Other_______________________
**Affiliation:**	Designation:<br><br>Institute Address:<br><br>Society membership:
**Contact number/s:**	
**E-mail address:**	
**Date of completion:**	_____/_______/______________<br>D D M M Y Y Y Y

INSTRUCTIONS FOR RECORDING RESPONSES:

1. Please respond to each item in the questionnaire.
2. Please consider applicability/availability/practices in GCC while selecting your response.
3. Please click the box that suggests your response.
4. A (☒) sign will appear in the box for responding to multiple choice queries once you click on it. Please ensure that the appropriate box is checked. Then, proceed to answer the next question. Multiple boxes can be selected in few questions, where indicated.
5. For descriptive answers, please type them in the space provided for comments.

1. What is the observed prevalence of PIK3CA, AKT1, and PTEN mutations among patients with hormone receptor-positive (HR+) human epidermal growth factor receptor 2-negative (HER2-) advanced breast cancer (aBC) in your clinical practice?

PIK3CA	☐ 0–20%	☐ 21–40%	☐ 41–60%	☐ 60–80%	☐ >80%
AKT1	☐ <5%	☐ 5–15%	☐ 15–25%	☐ 26–30%	☐ >30%
PTEN	☐ <5%	☐ 5–15%	☐ 15–25%	☐ 26–30%	☐ >30%
ESR1	☐ <5%	☐ 5–15%	☐ 15–25%	☐ 26–30%	☐ >30%

2. What is the percentage of co-mutations in your patient cohort?

PIK3CA + ESR1:	☐<5%	☐5-15%	☐15-25%	☐26-30%	☐>30%
PIK3CA + BRCA:	☐<5%	☐5-15%	☐15-25%	☐26-30%	☐>30%
AKT1 + ESR1:	☐<5%	☐5-15%	☐15-25%	☐26-30%	☐>30%
AKT1 + BRCA:	☐<5%	☐5-15%	☐15-25%	☐26-30%	☐>30%
PTEN + ESR1:	☐<5%	☐5-15%	☐15-25%	☐26-30%	☐>30%
PTEN + BRCA:	☐<5%	☐5-15%	☐15-25%	☐26-30%	☐>30%

3. Which guidelines do you follow for molecular/genetic testing recommendations for patients with HR+ HER2- aBC?

☐ NCCN
☐ ESMO
☐ National
☐ Other (please specify) ___

4. Which biomarker testing do you recommend after patients develop resistance to 1L among patients with HR+ HER2- aBC?

☐ PIK3CA
☐ AKT1
☐ PTEN
☐ ESR1
☐ Other (HER2mut, BRAF, NTRK, ROS1, etc.)
☐ All The Above
☐ Other (please specify) ___

5. Which testing platforms are preferred for assessing molecular markers in HR+ HER2- aBC?

☐ Next-Generation Sequencing (NGS)
☐ Polymerase Chain Reaction (PCR)
☐ Whole genome sequencing (WGS)
☐ Digital PCR
☐ Fluorescence in situ hybridization (FISH)
☐ Immunohistochemistry

6. How do you define primary and secondary endocrine resistance following progression on cyclin-dependent kinase 4/6 inhibitors (CDK4/6i) therapy?

Primary endocrine resistance

- ☐ Lack of response to first line (1L) endocrine therapy (ET) with progression within first 6 months of CDK4/6i
- ☐ Progression within first 12 months of ET regardless of CDK4/6i
- ☐ Failure to respond to any ET prior to or during CDK4/6i
- ☐ Progression within first 6 months of any ET
- ☐ Any of the above

Secondary endocrine resistance

- ☐ Initial response to ET with progression after 6 months of CDK4/6i
- ☐ Disease control for > 2 years of ET, with progression during CDK4/6i
- ☐ Progression after any period of disease control with ET combined with CDK4/6i
- ☐ All of the above
- ☐ Other (please specify)

____

7. What are the approximate percentages of patients experiencing primary and secondary endocrine resistance, as well as hormone-sensitive responses, following treatment for HR+ HER2- aBC in your practice?

Primary endocrine resistance:	☐10-20% ☐21-30% ☐31-40% ☐41-50% ☐>50%
Secondary endocrine resistance:	☐20-30% ☐31-40% ☐41-50% ☐51-60% ☐>60%
Hormone-sensitive:	☐30-40% ☐41-50% ☐51-60% ☐61-70% ☐>70%

8. When do you consider performing tissue biopsy and re-biopsy for biomarker testing among patients with aBC?

Biopsy	***Re-biopsy***
☐ At diagnosis<br>☐ At progression<br>☐ In both cases	☐ At metastasis<br>☐ At progression on 1L CDK4/6i + ET<br>☐ At suspected change in the cancer subtype (eg, from HR+ to triple-negative),<br>☐ All of the above

9. Which method and type of sample do you prefer when performing NGS after progression on 1L CDK4/6i + ET for HR+ HER2- aBC?

Preferred Method:	☐ Tissue biopsy ☐ Blood Sample for (ctDNA)
If tissue, Type of preferable tissue sample:	☐ Fresh ☐ Archived ☐ Either of them

10. If ctDNA testing for biomarker profiling initially yields negative results, would you consider proceeding with tissue-based next-generation sequencing (NGS) for confirmatory analysis or further investigation?

☐Yes, always

☐Yes, if clinical suspicion remains high

☐No, ctDNA results are sufficient

11. What are the approximate percentages of patients with HR+ HER2- aBC undergoing biomarker testing across different clinical cohorts?

***PI3K/AKT1/PTEN Testing*:**	☐0-20% ☐21-40% ☐41-60% ☐>60%
***ESR1 Testing*:**	☐0-20% ☐21-40% ☐41-60% ☐>60%
***BRCA Testing*:**	☐0-20% ☐21-40% ☐41-60% ☐>60%

12. What is the average duration of therapy with CDK4/6i as 1L treatment for patients with HR+ HER2- aBC?

☐6-12 months

☐1-3 years

☐3-5 years

☐>5 years

13. Do you consider CDK4/6i rechallenge in second-line (2L) treatment for HR+ HER2- aBC? If yes, for which patients, and what percentage of your patients does this apply to?

Rechallenge consideration:	☐ Yes ☐ No
Patient selection:	☐ Patients who previously responded to CDK4/6i for long time<br>☐ Patients with disease progression on several other lines of therapy<br>☐ Patients who do not have any identifiable biomarkers<br>☐ Patients with bone only metastasis<br>☐ Other (please specify) ____________________
Percentage of patients considered for rechallenge:	☐<10%<br>☐11-25%<br>☐26-40%<br>☐>40%

14. When do you consider switching a patient from hormonal-based regimens to chemotherapy or antibody-drug conjugates (ADCs)? (Select all that apply)

☐ Evidence of fast disease progression while on hormonal therapy

☐ Lack of adequate response to hormonal therapy after a defined treatment period

- ☐ Changes in biomarker status (eg, loss of HR expression or emergence of HER2 positivity)
- ☐ Patients with a high burden of disease (eg, multiple metastatic sites or large tumor sizes)
- ☐ Patients with metastasis in vital organs (eg, liver, lungs brain)
- ☐ All the above
- ☐ Other (please specify)

15. If fulvestrant is used before, do you consider using it in combination with other agents in a 2L endocrine-based regimen?

- ☐ Yes, it can be effectively combined with targeted therapies
- ☐ Yes, but only if there is evidence of partial response or sensitivity to hormonal therapy
- ☐ Yes, if there is long treatment-free interval after the initial use of fulvestrant
- ☐ No, switching to a different ET is preferred
- ☐ No, fulvestrant not preferred to be used in the 2L

16. Which factors are considered when introducing elacestrant to ESR1 mutant patients?

- ☐ Prior use of CDK4/6i for ☐ 6 months ☐12 months ☐18 months
- ☐ Presence of co-mutations (eg, PIK3CA, AKT1, PTEN, BRCA, HER2) that may affect treatment response
- ☐ Safety and tolerability considerations related to the patient's overall health
- ☐ Presence of visceral metastasis
- ☐ High burden of disease (eg, multiple metastatic sites, large tumor size)
- ☐ All of the above considerations

17. Which patient factors should be considered while deciding to use capivasertib in combination with fulvestrant?

- ☐ Patients with PIK3CA/AKT1/PTEN alterations
- ☐ Prior use of CDK4/6i for ☐<12 months ☐>12 months
- ☐ Presence of co-mutations (ESR1, BRCA)
- ☐ Safety considerations
- ☐ Presence of visceral metastasis (eg, liver)
- ☐ High burden of disease (eg, multiple metastatic sites, large tumor size)
- ☐ All of the above factors

18. If the following alterations are detected in a patient, which alteration would you consider targeting first?

Both BRCA and PIK3CA/AKT1/PTEN	***Both ESR1 and PIK3CA/AKT1/PTEN***	***Both BRCA and ESR1***
☐ BRCA mutations<br>☐ PIK3CA/AKT1/PTEN mutations<br>☐ Consider a combination approach targeting both alterations simultaneously	☐ ESR1 mutations<br>☐ PIK3CA/AKT1/PTEN mutations<br>☐ Consider a combination approach that addresses both alterations simultaneously	☐ BRCA mutations<br>☐ ESR1 mutations<br>☐ Consider a combination approach that addresses both alterations simultaneously
